# What is the role of theory in health behavior change interventions?

**DOI:** 10.1186/1479-5868-1-9

**Published:** 2004-07-23

**Authors:** Simone A French, Tony Worsley

**Affiliations:** 1 Division of Epidemiology, University of Minnesota, Minneapolis, MN, USA; 2 Centre for Physical Activity and Nutrition School of Health Sciences, Deakin University, Burwood Victoria, Australia

The discussion about the interplay between theory and intervention has a long history and much has been written and published. The companion perspective papers by Rothman and Jeffery provide alternative points of view on this important and complex question. Their papers are not intended to be critical reviews of the literature. Instead, the aims of the two papers are to delineate the problem, present the different perspectives of the authors on the issue, and suggest constructive steps researchers can take to move the field forward.

We hope that these articles generate a lively response from our readers and spur innovative ideas for strategies to cultivate a healthy developmental interplay between theory and intervention in behavioral nutrition and physical activity research.

We invite you to consider the intellectual and practical challenges posed by the two authors and perhaps respond with your perspectives, which may be published in the IJBNPA.

We look forward to hearing from you soon.

Simone A. French, Ph.D.

Tony Worsley, Ph.D.

Co-Editors, IJBNPA

